# Loss of Bmal1 impairs the glutamatergic light input to the SCN in mice

**DOI:** 10.3389/fncel.2025.1538985

**Published:** 2025-02-27

**Authors:** Hüseyin Korkmaz, Max Anstötz, Tim Wellinghof, Benedetta Fazari, Angelika Hallenberger, Ann Kathrin Bergmann, Elena Niggetiedt, Fatma Delâl Güven, Federica Tundo-Lavalle, Fathima Faiba A. Purath, Kevin Bochinsky, Lothar Gremer, Dieter Willbold, Charlotte von Gall, Amira A. H. Ali

**Affiliations:** ^1^Faculty of Medicine, Institute of Anatomy II, Heinrich Heine University, Düsseldorf, Germany; ^2^Core Facility for Electron Microscopy, Faculty of Medicine, Heinrich Heine University Düsseldorf, Düsseldorf, Germany; ^3^Jülich Research Center, Institute of Biological Information Processing (IBI-7: Structural Biochemistry), Jülich, Germany; ^4^Institute of Physical Biology, Heinrich-Heine-Universität Düsseldorf, Düsseldorf, Germany; ^5^Department of Human Anatomy and Embryology, Faculty of Medicine, Mansoura University, Mansoura, Egypt

**Keywords:** Bmal1, circadian system, SCN, vGLUT, GLAST, VIP, synapses

## Abstract

**Introduction:**

Glutamate represents the dominant neurotransmitter that conveys the light information to the brain, including the suprachiasmatic nucleus (SCN), the central pacemaker for the circadian system. The neuronal and astrocytic glutamate transporters are crucial for maintaining efficient glutamatergic signaling. In the SCN, glutamatergic nerve terminals from the retina terminate on vasoactive intestinal polypeptide (VIP) neurons, which are essential for circadian functions. To date, little is known about the role of the core circadian clock gene, Bmal1, in glutamatergic neurotransmission of light signal to various brain regions.

**Methods:**

The aim of this study was to further elucidate the role of Bmal1 in glutamatergic neurotransmission from the retina to the SCN. We therefore examined the spontaneous rhythmic locomotor activity, neuronal and glial glutamate transporters, as well as the ultrastructure of the synapse between the retinal ganglion cells (RGCs) and the SCN in adult male Bmal1−/− mice.

**Results:**

We found that the deletion of Bmal1 affects the light-mediated behavior in mice, decreases the retinal thickness and affects the vesicular glutamate transporters (vGLUT1, 2) in the retina. Within the SCN, the immunoreaction of vGLUT1, 2, glial glutamate transporters (GLAST) and VIP was decreased while the glutamate concentration was elevated. At the ultrastructure level, the presynaptic terminals were enlarged and the distance between the synaptic vesicles and the synaptic cleft was increased, indicative of a decrease in the readily releasable pool at the excitatory synapses in Bmal1−/−.

**Conclusion:**

Our data suggests that Bmal1 deletion affects the glutamate transmission in the retina and the SCN and affects the behavioral responses to light.

## 1 Introduction

In addition to its important function in image perception, light has a strong influence on a variety of brain functions. This includes the synchronization of rhythmic brain and body functions to the external time but also mood, emotions and cognition (Mahoney and Schmidt, 2024). Therefore, lighting conditions that deviate from the natural light-dark cycle can lead to chronodisruption and alter various brain functions (von Gall, 2022). On the other hand, chronodisruption might be linked to neurodegenerative diseases (Musiek and Holtzman, 2016). Rhythmic brain and body functions controlled by the circadian clock in the hypothalamic suprachiasmatic nucleus (SCN) can be entrained to rhythms in the environment such as the light-dark cycle. The light-dark cycle is also capable of modulating behavior and physiology independent of the circadian clock in a process called “masking” (Mrosovsky, 1999).

Intrinsically photosensitive retinal ganglion cells (ipRGCs), which contain the photopigment melanopsin, are essential for photic entrainment (Hattar et al., 2002; Panda et al., 2002; Ruby et al., 2002; Berson et al., 2002) and masking (Mrosovsky and Hattar, 2003) and mediate various other brain function responses to light, including alertness/sleep, mood/emotion and cognition (Lazzerini Ospri et al., 2024; LeGates et al., 2014; Zhang et al., 2021; Fernandez et al., 2018). A recent study by Lazzerini Ospri et al. (2024) highlights the importance of light mediated by the ipRGC for the integrity, activity and function of neural networks involved in the regulation of social, emotional and cognitive states. The SCN receives almost exclusively input from ipRGC (Beier et al., 2021) whose fibers form the retino-hypothalamic tract and mainly reach the vasoactive intestinal polypeptide (VIP)-containing neurons in the core region of the SCN (Kim et al., 2019). The VIP-containing SCN neurons are activated by light and are crucial for light entrainment (Jones et al., 2018; Todd et al., 2020). VIP also contributes significantly to maintain the circadian rhythm in the SCN at the cellular and network levels (Hamnett et al., 2019).

Glutamate represents the dominant neurotransmitter of the retina for image formation (Boccuni and Fairless, 2022) and non-image formation (Purrier et al., 2014). The neuropeptide pituitary adenylyl cyclase-activating peptide (PACAP) released as a co-transmitter by retinal ganglion cells, appears to be dispensable for masking by light (Colwell et al., 2004). The synaptic strength of synapses essentially depends on the synaptic vesicles. In glutamatergic synapses, the vesicular glutamate transporters vGLUT1 and vGLUT2 appear to be crucial not only for packing but also for vesicle trafficking and synaptic efficacy (Daniels et al., 2004; Engelund et al., 2010; Wojcik et al., 2004) with distinct synaptic release sites and in a non-overlapping complementary manner (Fremeau et al., 2001; Fremeau et al., 2004). Moreover, the glutamate transporters show a light-dependent oscillation in the brain which seems to be dependent on the molecular clockwork (Yelamanchili et al., 2006; Kiss et al., 2007; Darna et al., 2009; Chi-Castañeda and Ortega, 2018). In the SCN, the diurnal difference in the vGLUT1 and vGLUT2 immune response can be observed particularly in the area of the VIP neurons, which suggests a selective synaptic remodeling at sites of photic integration (Girardet et al., 2010). In addition, astrocytes contribute to the tripartite synapse with perisynaptic processes and regulate the extracellular glutamate level by both glutamate release and precise glutamate reuptake. The astrocytic glutamate aspartate transporter (GLAST) is essential for glutamate reuptake, thereby also preventing excitotoxicity (Todd and Hardingham, 2020), which is implicated in the pathogenesis of neurodegenerative diseases (Cuellar-Santoyo et al., 2023). Interestingly, VIP appears to be involved in preventing neurotoxicity in the brain by increasing glutamate reuptake (Brown, 2000), presumably through an increase in GLAST activity mediated by the VIP/VPAC2 receptor (Goursaud et al., 2008). Astrocytic glutamate reuptake and glutamine synthetase activity show daily rhythms (Leone et al., 2015). The astrocytic molecular clockwork appears to modulate GLAST and thereby the extracellular glutamate concentration (Beaulé et al., 2009; Cagampang et al., 1996; Spanagel et al., 2005). In the SCN, astrocytes contribute to the circadian timekeeping by modulating GABA- (Barca-Mayo et al., 2017) and glutamatergic-signaling (Chi-Castañeda and Ortega, 2018; Brancaccio et al., 2019).

Rhythmic cell function is controlled by a molecular clockwork which is composed of autoregulatory transcriptional/translational feedback loops of clock genes. The two transcription factors, Bmal1 and Clock are essential components of the molecular clockwork (Reppert and Weaver, 2002). In mice, Bmal1 deletion leads to a complete loss of circadian rhythmicity in constant darkness (Bunger et al., 2000) highlighting the importance of this clock gene for the circadian clock. Bmal1-deficient mice (Bmal1−/−) mice show a variety of pathological structural and functional changes throughout the body and brain as a result of accelerated aging, which is probably due to impaired redox homeostasis (Kondratov et al., 2006; Musiek et al., 2013; Ali et al., 2015). However, young Bmal1−/− mice also show morphological changes in perisynaptic astrocytic processes, suggesting a role of the molecular clockwork in the integrity of the tripartite synapse (Ali et al., 2020). Although Bmal1−/− mice show masking, their activity rhythm in the light-dark cycle is less robust than in their wild-type littermates (Pfeffer et al., 2015; Bunger et al., 2000) and the light-induction of clock genes is impaired (Pfeffer et al., 2009). Moreover, retina-specific deletion of Bmal1 affects visual image processing (Baba et al., 2018). These findings indicate an important role for Bmal1 in the transmission of light information from the retina to the brain. However, so far little is known about the role of Bmal1 in glutamatergic neurotransmission.

The aim of this study was to further elucidate the role of Bmal1 in glutamatergic neurotransmission from the retina to the brain. We therefore examined the neuronal and glial glutamate transporters as well as the ultrastructure of the synapse between the retina and the SCN, in adult Bmal1−/− mice. We focused on the SCN core region as an example because it represents the best characterized structure for ipRGC input. Our study contributes to a better understanding of the role of molecular clockwork in processing non-image-forming light information in the brain, which is also relevant in the context of changes in mood/emotions and cognition due to chronodisruption.

## 2 Material and methods

### 2.1 Experimental animals

Bmal1−/− and Bmal1+/+ littermates were obtained from breeding of Bmal1 heterozygous (Bmal1+/−) mice. Initial breeding pairs were obtained from Jackson Laboratories (B6.129-Arntltm1Bra/J). Adult male mice aged 4 months were used in this study (total: *n* = 19 Bmal1+/+, *n* = 20 Bmal1−/−). Mice were housed in standard cages in a temperature-controlled chamber (Beast Master, Germany) at the local animal facility of Düsseldorf University Hospital under 12-h light and 12-h darkness (lights on at 6:00 AM and off at 6:00 PM). The illumination (white light) intensity was 600 lx at the light phase and 0 lx during the dark phase. The mice had free access to food and water *ad libitum*.

All animal experiments were approved by the North Rhine-Westphalia State Agency for Nature, Environment and Consumer Protection (LANUV), Germany (approval number: 84.02.04.2012 A102 and 84-02.04.2014 A314) and conform to international guidelines for the ethical use of experimental animals (Portaluppi et al., 2010).

### 2.2 Monitoring of spontaneous locomotor activity

Bmal1−/− and Bmal1+/+ littermates (*n* = 6 mice per genotype) were housed individually in standard cages. Spontaneous locomotor activity was continuously recorded using on-cage infrared detectors (Mouse-E-Motion, Hamburg, Germany). The activity counts and the amplitude of the 24-h rhythm in spontaneous locomotor activity were analyzed using Clocklab software (Actimetrics, Wilmette, IL, USA) (Öztürk et al., 2021; Hassan et al., 2021). The percentage of activity counts during the day and the night to the total activity counts was calculated.

### 2.3 Immunohistochemistry

#### 2.3.1 Tissue preparation

Mice (*n* = 5 mice per genotype) were sacrificed around the middle of the light phase (12: 00 pm = ZT06) by an overdose of ketamine/xylazine mixture (100 mg/kg or 10 mg/kg body weight, respectively), injected intraperitoneally. Mice were transcardially perfused with ice-cold 0.9% NaCl followed by 4% formalin in 0.1 M phosphate-buffered saline (PBS) using a Ministar Peristaltic Pump (World Precision Instruments, Sarasota, FL, USA). The eyes and the brains were carefully dissected, post-fixed in 4% formalin in PBS for 4 and 24 h, respectively. For cryoprotection, eyes and brains were stored subsequently in 20% and 30% sucrose for 24 h each and then embedded with optimum cutting temperature (OCT) compound. The eyes were cut into 14 μm thick sections along the anteroposterior axis, while the brains were cut into 20 μm thick coronal sections in the rostro-caudal extent of the SCN using a cryostat (Leica CM, Wetzlar, Germany).

For immunohistochemistry of whole-mount retina, mice (*n* = 4 mice per genotype) were killed using isoflurane. The eyes were dissected, fixed with 4% formalin for 15 min then washed with PBS. The anterior segment of the eye was removed, and the retina was separated from the sclera and the vitreous body. The retina was incised at the edges and dripped with cold methanol to smooth it out.

#### 2.3.2 H&E staining

Retinal sections were rehydrated with Roticlear, followed by a descending alcohol series (100, 96, and 70%) and finally with distilled water. Then, the sections were stained with a Mayer’s Hemalum solution (FA Merck 1.09249.1000) for 20 s and rinsed with 0.1% acid ethanol for 15 s followed by rinsing with tap water for 3–5 min. Sections were stained with Erythrosine B solution 0.1% aqueous for 1.5 min and rinsed again with distilled water for 30 s. Sections were dehydrated using increasing alcohol gradient (70, 96, and 100% EtOH) followed by Rothistol each twice for 5 min then cover-slipped using DEPEX.

#### 2.3.3 Immunofluorescence

Slides with retinal and SCN sections were rinsed with 0.3% triton X-100 in PBS (PBST) and then incubated in 5% normal goat serum, in PBST and 0.25% BSA for 1 h at RT to quench unspecific antibody binding. Parallel retinal and brain sections were incubated with guinea pig anti-vGLUT1 (1:4000, Synaptic Systems, cat. #135304, Göttingen, Germany) or rabbit polyclonal anti-vGLUT2 antibody (1:3000, Synaptic Systems, cat. #135402 Göttingen, Germany). Additional parallel brain sections were incubated with rabbit polyclonal anti-VIP antibody (1:250, Acris/Peninsula Lab Burlington, MA, USA), rabbit polyclonal anti- EAAT1/GLAST-1 antibody (1:1000, ab416 Abcam Cambridge, Great Britain) or mouse monoclonal anti-GFAP antibody (1:500 BD-Pharmingen TM Material Number 556330, Franklin Lakes, New Jersey, USA) overnight at 4°C. Slides were then rinsed with PBST and subsequently incubated with the corresponding secondary antibodies: goat anti-mouse Alexa Fluor^®^568, goat anti-rabbit Alexa Fluor^®^488, goat anti-rabbit Alexa Fluor^®^568, goat anti-guinea pig Alexa Fluor^®^488 (1:500, Molecular Probes, Eugene, OR, USA) for 1 h at RT. Slides were rinsed and then cover-slipped with the mounting medium (Fluoromount antifade, Southern Biotech). Retina whole-mounts were stained free floating using rabbit polyclonal anti-melanopsin (1:500, Clone UF008, cat#:1:AB-N39, Advanced Targeting Systems, San Diego, CA, USA) followed by goat anti-rabbit Alexa Fluor^®^488 and cover slipped as mentioned above.

#### 2.3.4 Image acquisition

Images of H&E-stained retinal sections were acquired in bright field mode using 40× objective. Z-stacks of immunofluorescence images of melanopsin-stained whole mount retina and single immunofluorescence images of VIP-stained SCN were acquired using a 20× objective of fluorescence microscope (KEYENCE BZ 900E, Keyence Corporation, Osaka, Japan), equipped with the respective fluorescence filters and lenses.

Single images of vGLUT1-, vGLUT2- and GLAST-immunofluorescence in the SCN were acquired using a confocal microscope (LSM SP8, Leica, Germany), equipped with high-resolution 63× NA 1.3 objective lenses Leica dye assistant was used and line sequential acquisition was selected. The following settings were used: format 1024×1024, zoom factor 4, a pinhole size of 1 Airy unit and a speed of 200. Confocal photomicrographs were acquired from 3 independent fields within the SCN core region on both sides. The microscope acquisition settings were kept consistent in all samples for each staining set.

#### 2.3.5 Image analysis

The genotype was obscured to the investigators. Image analysis of H&E staining and immunofluorescence images of retina and VIP was performed using the BZ-II analyzer software (Keyence Corporation, Osaka, Japan). Intact retinal sections devoid of histological artifacts were used. The width of the individual retinal layers at the midpoint between the optic disk and peripheral end of the retina was measured in three HE-stained retinal sections and averaged per animal. The Z-stacks of immunofluorescence images of melanopsin-stained whole mount retina were merged, the number of melanopsin positive retinal ganglion cells was counted manually in four independent fields in whole mount retina, which involves all retinal layers, and the density (cells/mm^2^) was calculated. Background corrected fluorescence intensities in arbitrary units (a.u.) of the immunoreaction for the vesicular glutamate transporter (vGLUT1- and vGLUT2-Ir), GLAST-Ir and of VIP-Ir were analyzed in three parallel sections of each mouse and averaged. The background corrected fluorescence intensities (in a.u.) and the number of vGLUT1-, vGLUT2- and GLAST- positive particles were analyzed within the SCN core region using ImageJ software.^1^ Only sections without artifacts or big blood vessels were included in the analysis. The particle analysis was performed in a defined area (46.18 μm × 46.18 μm) using macro including a median filter with a radius of 2 pixels, background subtraction using a rolling ball with a radius of 15 pixels, edges were enhanced using an unsharp mask (radius = 4, mask weight = 0.60) and threshold (38, 255). For particle separation, the image was converted to binary mask and the touching particles were separated by watershed.

### 2.4 Postmortem SCN neurochemistry

#### 2.4.1 Tissue preparation and dual-phase metabolite extraction

Seven Bmal1+/+ and eight Bmal1−/− littermates were killed by cervical dislocation during the light phase (ZT6-ZT8). The brains were quickly dissected and cut into 1 mm thick sections in the coronal plane using an ice-cold mouse brain matrix. The SCN was bilaterally microdissected using a punch needle (15G), weighed and snap frozen. SCN tissue was homogenized in ice-cold double-distilled water (ddH_2_O) and subjected to methanol/chloroform/water (1:1:1, v/v/v) dual-phase extraction as previously described (Koch et al., 2016). Briefly, 1 ml of ice-cold methanol was then added to the homogenized tissue, vortexed vigorously and incubated on ice for 15 min. Then 1 ml of ice-cold chloroform was added, vortexed and incubated for 10 min on ice. Finally, 800 μl of chilled ddH_2_O were added, vortexed and incubated at 4°C overnight to enable phase separation. On the following day, the samples were centrifuged at 4,500 rpm for 25 min at 4°C. The upper phase containing the water-soluble metabolites was carefully separated. Lyophilization was then performed, and samples were stored at −80°C until measurement.

#### 2.4.2 Proton nuclear magnetic resonance (^1^H-NMR) spectroscopy

Lyophilized tissue extracts obtained from dual phase metabolite extraction were resuspended in 210 μl 20 mM phosphate buffer (pH 7.0) containing 10% D_2_O and 3-(Trimethylsilyl) propanoic acid (TSP; Lancaster Synthesis) as internal standard and transferred into 3 mm standard NMR tubes (Norell).

^1^H NMR spectra of extracts were acquired on a 700 MHz (16.4 Tesla) Bruker AVANCE III HD 700 spectrometer equipped with a TCI 700S3 H-C/N-D-05 Z cryoprobe (Bruker). NMR data using 1-dimensional ^1^H-experiments with excitation sculpting as water suppression were obtained with the following acquisition parameters: 25°C sample temperature, sweep width of 14 ppm, acquisition time 2 sec, relaxation delay 10 sec.

Metabolic glutamate determination was obtained by integration of ^1^H NMR signals in the range of 2.3776–2.3386 ppm (Supplementary Figure 1A) characteristic for the CγH_2_ moiety of glutamate and concentrations were calculated using the ERETIC2 concentration calculation tool from TopSpin (TopSpin software package, Vers. 3.6.4, Bruker). Glutamate standards were used as external standards and standard control measurements confirmed linearity of integrated signal intensity in the tested concentration range (glutamate concentrations of 95–526 μmol/L in the NMR test tube) (Supplementary Figure 1B).

### 2.5 Focused ion beam scanning electron microscopy

Mice (*n* = 3 per genotype) were killed by an overdose of ketamine/xylazine mixture as described in section “2.3.1 Tissue preparation.” Mice were then transcardially perfused with 0.1 M phosphate buffer followed by the ice-cold 0.15 M cacodylate buffer with 2% formalin, 2.5% glutaraldehyde and 2 mM CaCl_2_. Brains were dissected, post-fixed for 2 h at 4°C in the same fixative then rinsed using cacodylate buffer. Coronal brain sections of 150 μm thickness through the SCN between Bregma −1.5 and −2 (Paxinos and Franklin, 2012) were cut using a vibratome (VT1000S; Leica Microsystems GmbH, Wetzlar, Germany). Sections containing the mid-SCN level were selected from each mouse for further processing. Sections were rinsed with ice-cold 0.15 M cacodylate buffer containing 2 mM CaCl_2_ and then incubated with cacodylate buffer containing 2% OsO_4_ and 1.5% potassium ferrocyanide for 1 h. Sections were rinsed with ddH_2_O and then incubated with 1% thiocarbohydrazide for 25 min at RT. Sections were rinsed with ddH_2_O followed by incubation with 2% aqueous OsO_4_ for 30 min at RT. After subsequent washing, sections were incubated with 1% aqueous uranyl acetate at 4°C overnight. Then, sections were rinsed and incubated with pre-warmed 0.02 M lead nitrate in 0.03 M aspartic acid, after adjusting the pH to 5.5, for 30 min at 60°C.

After washing with ddH_2_O, sections were dehydrated in an ascending series of ethanol (30%, 50%, 60%, 70%, 90%, 95% to 100%) each 10 min followed by transferred to propylene oxide twice each for 2 min, then, to an ascending mixture of propylene oxide and epoxy resin (2:1, 1:1, 1:2) each for 1 h (Durcupan™; ACM, Fluka, Neu-Ulm, Germany). Finally, sections were transferred to pure epoxy resin and left overnight in RT. Then, sections were flat embedded in fresh epoxy resin within silicone rubber embedding molds and the epoxy resin blocks were allowed to polymerize at 60°C for two days.

Epoxy resin blocks were trimmed around the tissue specimen containing SCN, semi-thin sections (100 nm) were cut on an ultramicrotome (Leica Ultracut UCT, Leica Microsystems GmbH, Wetzlar, Germany) and stained with Richardson’s stain. The sample was attached to a sample stub using conductive silver paste (Cat# S066, Mikrotechnik Dr. Hert GmbH, Munich, Germany) and all the surfaces, except the surface to be imaged, were covered with silver paste to avoid charging of the epoxy-resin (Merchán-Pérez et al., 2009). The sample was left to dry overnight and then sputter-coated with a 20–24 nm thick layer of gold (108 auto, Cressington Scientific Instruments, Watford, UK).

Core region of SCN was identified by bright field microscopy as the area of interest and correlated with the optic image of the corresponding block surface in Focused Ion Beam Scanning Electron Microscope (FIB-SEM) (Crossbeam 550, Carl Zeiss AG, Oberkochen, Germany). FIB-SEM was used as it allows high resolution serial imaging simultaneously with sample surface removal using a focused gallium ion beam on a nanometer scale. The region of interest within core region of SCN was protected from undesired beam damage by a layer of platinum followed by a layer of carbon. This protective layer also contains tracking marks for automated image acquisition. Then, a trench, as a viewing channel, was coarsely milled using the FIB using 30 kV/15 nA ion beam current. The exposed cross section surface was further fine polished with the FIB using 30 kV/1.5 nA ion beam current.

The cross-section surface was serially milled using 30 kV/300 pA–30 kV/1.5 nA ion beam current depending on the sample and imaged with SEM perpendicular to the cutting plane using 2 kV acceleration voltage and an electron beam current of 69 pA with a frame time of 1 min 33 s per milling/imaging cycle. The backscattered electrons were detected by an InLens and an SE2 detector at a resolution of 2 nm/pixel and a slice thickness of 6 nm. A volume of 10 μm × 10 μm × 10 μm of dorsal SCN was imaged. The Atlas 5 software (Carl Zeiss AG, Oberkochen, Germany) was used for the collection of stacks of SEM images and automated correction of focus and alignment/astigmatism of serial micrographs. ImageJ software^1^ was used for image pinning.

Six to seven presynaptic ends per animal were used for quantitative analysis. The presynaptic terminals were further classified based on the appearance of post synaptic density (PSD) into asymmetric synapses with a PSD thicker than the presynaptic membrane, while symmetric synapses have a similar PSD width to the presynaptic membrane. Asymmetric and symmetric synapses are correlated to excitatory or inhibitory synapses, respectively (Romaus-Sanjurjo et al., 2021). It has been previously shown that RHT synapses in the SCN contain both small clear core vesicles (glutamate-releasing) and dense core vesicles (PACAP-releasing) (Hannibal et al., 2000). Thus, only the excitatory presynaptic terminals containing both small clear core vesicles (40–60 nm in diameter) and larger dense core vesicles (80–100 nm in diameter), of asymmetric synapses in core region of SCN were included. The presynaptic terminal was delineated in serial images with a step size of 90 nm (15 image interval, each 6 nm). The surface area of the delineated presynaptic terminal and the area of synaptic contact were determined in each plane. The synaptic vesicles were counted in the labeled planes and their sum represented the number of vesicles in each presynaptic terminal. For each synapse, the density of synaptic vesicles per μm^2^ was calculated and averaged for each animal. The volume of the presynaptic terminal was calculated by multiplying the measured presynaptic terminal area in subsequent planes by the step size. We further analyzed the mean distance of the synaptic vesicles to the synaptic cleft by averaging the minimal Euclidean distance of each vesicle to the associated reconstructed post synaptic density (PSD). This was used as an indicator of the readily releasable pool (RRP), defined as a subset of the vesicles in a presynaptic bouton that is more readily released than other vesicles and thus, closer to the presynaptic membrane. The reconstruction of vesicle positions and the PSD was performed in ImageJ. Subsequent calculation of the minimal Euclidean distances was performed in Microsoft Excel.

### 2.6 Statistical analysis

Statistical analysis was performed using Graph Pad Prism software. Values are presented as mean ± SEM. The normal distribution of the values within the groups of each data set was tested by Shapiro–Wilk test. When the values passed the normality test, parametric tests such as One-way ANOVA followed by Tukey’s *post-hoc* test for multiple group comparisons or the Student *t*-test for comparison between two groups were applied. When the values were not normally distributed, non-parametric test, the Mann–Whitney-U test was applied. *p* < 0.05 was considered statistically significant.

## 3 Results

### 3.1 Bmal1 deficiency affects the rhythm in spontaneous locomotor activity under a light/dark cycle

**FIGURE 1:**
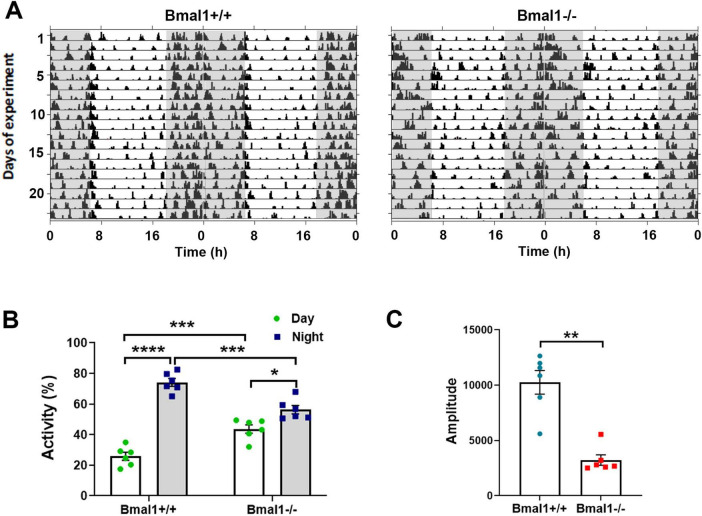
Bmal1 deficiency affects the rhythmic spontaneous locomotor activity. **(A)** Representative double plotted actograms of spontaneous locomotor activity of Bmal1+/+ and Bmal1–/– mice under a 12 h light/12 h dark cycle (LD). Gray-shaded boxes indicate periods of darkness. **(B)** Quantification of activity percentage during the light phase (white bars) and the dark phases (gray bars) to the total activity under the LD. One-way ANOVA test followed by Tukey’s multiple comparisons test. **(C)** Amplitude of the 24 h rhythm in spontaneous locomotor activity. Mann–Whitney U-test. Values are shown as mean + SEM. *p < 0.05, **p < 0.01, ***p < 0.001. ****p < 0.0001. *n* = 6 mice per genotype.

The spontaneous locomotor activity was monitored under standard photoperiod of 12 h light/12 h darkness to study the response to light. The total activity count during a 24 h period was not significantly different between both groups. Both genotypes showed a higher activity during the dark phase than during the light phase (Figures 1A, B). However, Bmal1−/− mice showed a significantly lower activity during the dark phase and a significantly higher activity during the light phase compared to the Bmal1+/+ (Figure 1B). Moreover, the amplitude of the 24 h period was significantly lower in Bmal1−/− mice compared to Bmal1+/+ mice (Figure 1C). These findings indicate an impaired response to the light/dark cycle.

### 3.2 Bmal1 deficiency affects retinal morphology but not the number of ipRGCs

**FIGURE 2:**
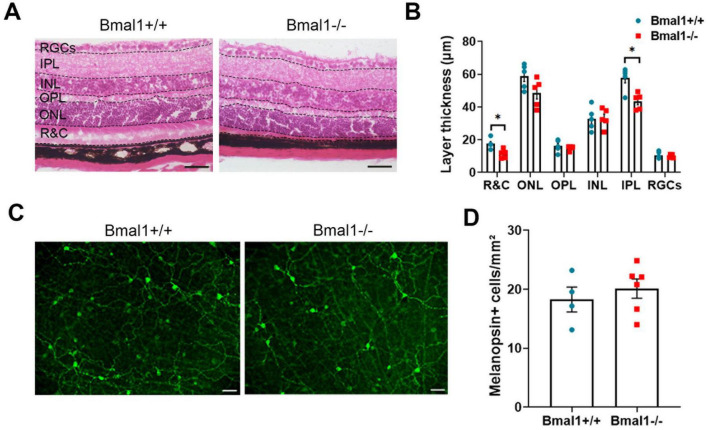
Bmal1 deficiency affects retinal morphology. **(A)** representative photomicrographs showing Hematoxylin and Eosin (HE)-stained retinal sections from Bmal1+/+ and Bmal1–/– mice. **(B)** Quantitative analysis of the thickness of retinal layers using Mann–Whitney-U-Test. R&C, rods and cones layer; ONL, outer nuclear layer; OPL, outer plexiform layer; INL, inner nuclear layer; IPL, inner plexiform layer; RGC, retinal ganglion cells. **(C)** Retinal whole-mounts showing melanopsin-immunoreactive cells (green) indicative for intrinsically photosensitive retinal ganglion cells (ipRGCs). **(D)** Quantification of the number of melanopsin positive (+) cells in the retina. Scale bars = 50 μm. Unpaired *t*-test, *P < 0.05.

As the light information is received by the retina, we investigated the retinal architecture in HE-stained sections. Although retinal layering was preserved, the rods and cones layer as well as the inner plexiform layer were significantly thinner in Bmal1−/− mice than in wildtype littermates (Figures 2A, B). The number of melanopsin+ cells was not different between Bmal1+/+ (18.3 ± 2.1 cells/mm^2^) and Bmal1−/− mice (20.1 ± 1.6 cells/mm^2^) (Figures 2C, D). This indicates that the number of ipRGCs is not affected by Bmal1 deficiency.

### 3.3 Bmal1 deficiency affects the vesicular glutamate transporters in the retina

Since glutamate is the predominant neurotransmitter in the retina, we analyzed the vesicular glutamate transporters, vGLUT1 and vGLUT2 in retinal layers.

**FIGURE 3:**
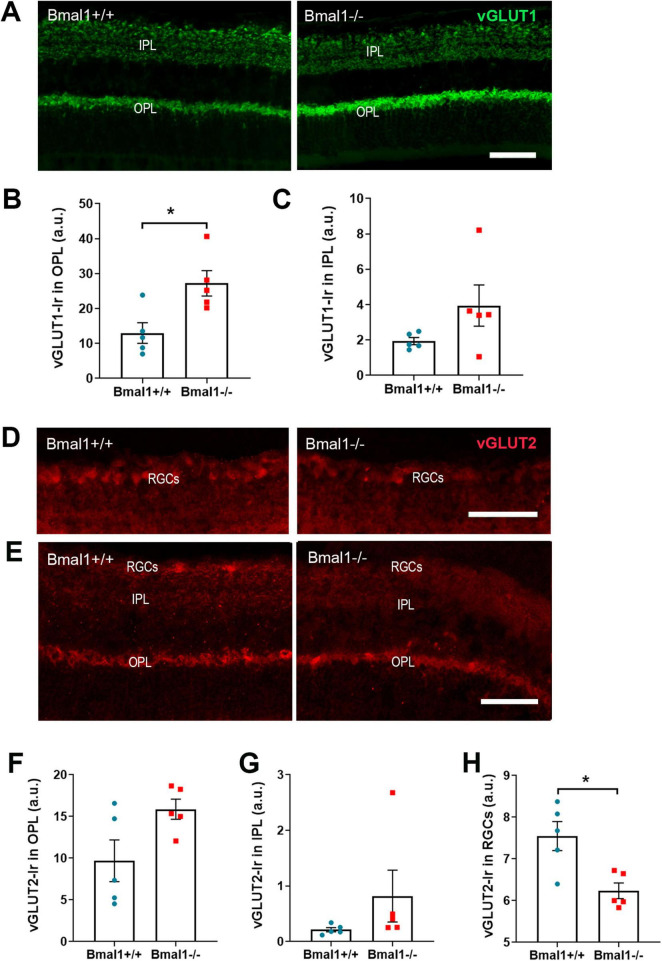
Bmal1 deficiency affects the vesicular glutamate transporters (vGLUT) in the retina. **(A)** Representative photomicrographs of vGLUT1-immunoreaction (green) in retinal sections of Bmal1+/+ and Bmal1–/– mice. Quantitative analysis of immunoreaction (Ir) of vGLUT1 in the **(B)** OPL and **(C)** IPL. **(D,E)** Representative photomicrographs of vGLUT2-Ir (red) in retinal sections from Bmal1+/+ and Bmal1–/– mice. Quantification of vGLUT2-Ir in the **(F)** OPL, **(G)** IPL and **(H)** RGCs. Scale bars = 50 μm. Unpaired-*t*-test, *P < 0.05.

vGLUT1-immunoreaction (Ir) was present in OPL and IPL (Figure 3A). In the OPL, vGLUT1-Ir was significantly lower in Bmal1+/+ mice than in Bmal1−/− mice (Figure 3B). In the IPL, vGLUT1-Ir was not significantly different between the genotypes (Figure 3C).

vGLUT2-Ir was present in IPL, OPL, and RGCs (Figures 3D, E). vGLUT2-Ir was not significantly different between the genotypes in the OPL (Figure 3F) and IPL (Figure 3G). However, in the RGCs, vGLUT2-Ir was significantly higher in Bmal1+/+ mice than in Bmal1−/− mice (Figure 3H). This indicates that Bmal1 deficiency alters the vGLUT1, 2 in retinal layers.

### 3.4 Bmal1 deficiency affects the vesicular glutamate transporter in the SCN core region

As glutamate is the main neurotransmitter that mediates light information from the retina to the brain, we exemplarily examined the vesicular glutamate transporters in the retinorecipient core region of the SCN.

**FIGURE 4:**
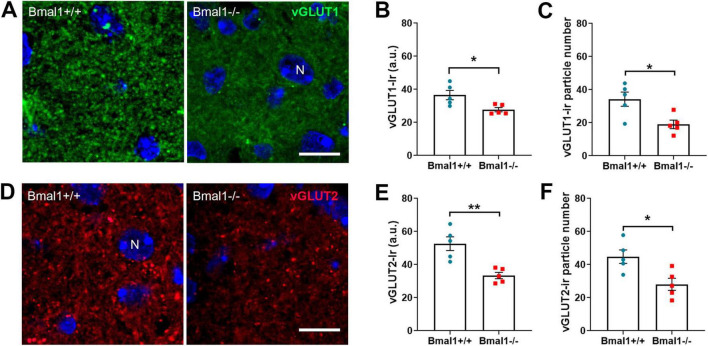
Bmal1 deficiency affects the vesicular glutamate transporters in the core region of the suprachiasmatic nucleus (SCN). **(A)** representative photomicrographs of vGLUT1-immunoreaction (green) in the SCN core region of Bmal1+/+ and Bmal1–/– mice. **(B)** Quantification of the fluorescent vGLUT1-immunoreaction (Ir) in arbitrary units (a.u.). **(C)** Number of vGLUT1-immunoreactive particles per SCN. **(D)** Representative photomicrographs of vGLUT2-Ir (red) in SCN core region of Bmal1+/+ and Bmal1–/– mice. **(E)** Quantification of fluorescent vGLUT2-Ir. **(F)** Quantification of the number of vGLUT2-immunoreactive particles. Immunoreactive particles were analyzed in a 46.18 μm^2^ area. N, DAPI-stained cell nuclei (blue). Scale bars = 10 μm. Unpaired-*t*-test, *P < 0.05, **P < 0.01.

In the core region of the SCN, vGLUT1-Ir was higher in Bmal1+/+ mice than in Bmal1−/− mice (Figures 4A–C). Similarly, vGLUT2-Ir was higher in Bmal1+/+ mice than in Bmal1−/− mice (Figures 4D–F), indicating that Bmal1 decreases the vGLUT1, 2 in SCN.

### 3.5 Bmal1 deficiency affects the ultrastructure of presynaptic terminals in SCN core region

Next, we analyzed the integrity of the presynaptic terminals in the SCN core region using Focused Ion Beam-Scanning Electron Microscopy (FIB-SEM).

**FIGURE 5:**
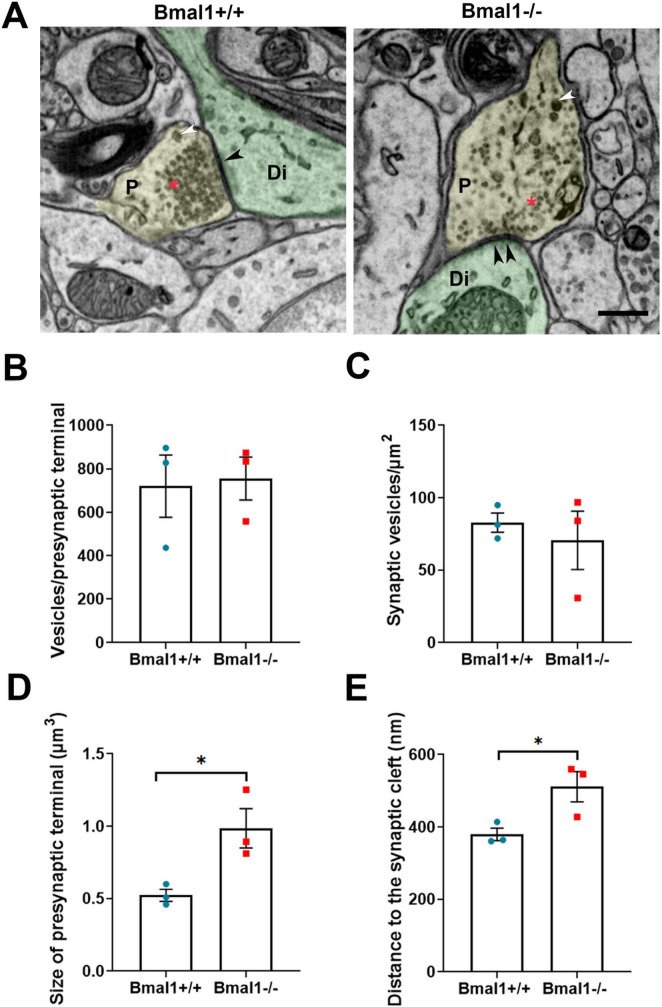
Bmal1 deficiency affects the ultrastructure of presynaptic terminals of the asymmetric/excitatory synapses in SCN core regions. **(A)** Representative electron micrographs showing presynaptic terminals (P, yellow), postsynaptic dendrites (Di, green) and synaptic density (black arrowhead) of asymmetrical synapses in SCN core region in Bmal1+/+ and Bmal1–/– mice. The red asterisk shows the small clear core vesicles; the white arrowhead shows the large dense core vesicles. Scale bar = 500 nm. **(B)** Quantification of the number of synaptic vesicles pro presynaptic terminal. **(C)** Quantification of synaptic vesicles density/μm^2^ at the presynaptic terminal. **(D)** Quantification of the size of the presynaptic terminals in μm^3^. **(E)** Quantification of the mean distance between the synaptic vesicles and the synaptic cleft in nm. Unpaired-*t*-test, *n* = 3 mice per genotype. *P < 0.05.

In asymmetric, and therefore putative excitatory, synapses (Figure 5A), the number and the density of vesicles in the presynaptic terminals was not different between both genotypes (Figures 5B, C). However, in Bmal1−/− mice the size of presynaptic terminals (Figure 5D) as well as the distance of the vesicles to the synaptic cleft (Figure 5E) was larger compared to Bmal1+/+ mice.

In symmetric, and therefore putative inhibitory, synapses, there were no significant differences between the genotypes in the number and density of synaptic vesicles, in the size of the presynaptic terminals or in the distance of the vesicles to the synaptic cleft (Supplementary Figure 2). This data suggests that only excitatory, but not inhibitory synapses are altered in Bmal1−/− mice.

### 3.6 Bmal1 deficiency affects glutamate reuptake in the SCN

**FIGURE 6:**
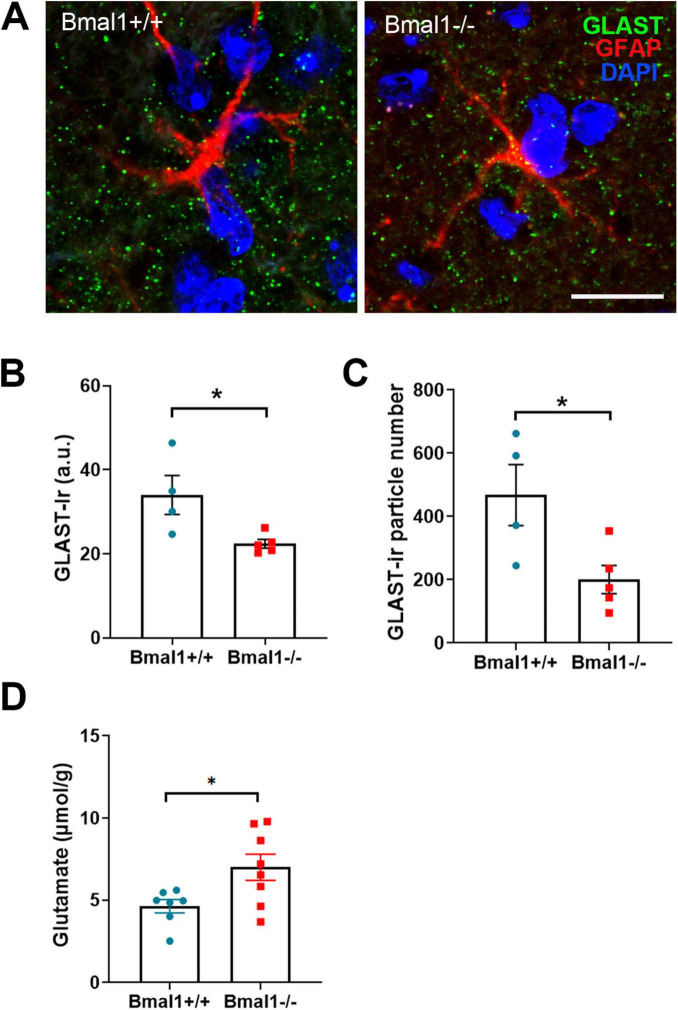
Bmal1 deficiency affects the glial glutamate transporter GLAST and glutamate levels in the suprachiasmatic nucleus (SCN). **(A)** representative photomicrographs showing immunoreaction of GLAST (green), astrocyte stem processes marker GFAP (red) and DAPI-stained nuclei (blue) in SCN core region in Bmal1+/+ and Bmal1–/– mice. Scale bar = 10 μm. **(B)** Quantification of immunoreaction (Ir) of GLAST in the SCN core in arbitrary unit (a.u.). **(C)** Quantification of the number of GLAST-Ir particles per SCN field (46.18 μm × 46.18 μm). **(D)** Quantification of postmortem glutamate concentration in the SCN. Unpaired-*t*-test, *P < 0.05.

As the glial glutamate transporter GLAST is essential for the reuptake of glutamate from the synaptic cleft, we analyzed GLAST in the SCN core region. GLAST-Ir was significantly higher in Bmal1+/+ than in Bmal1−/− mice (Figures 6A–C). Moreover, the glutamate concentration was lower in SCN extracts from Bmal1+/+ than in those from Bmal1−/− mice (Figure 6D). These findings suggest an impaired glial reuptake of glutamate from the synaptic cleft in the SCN of Bmal1−/− mice.

### 3.7 Bmal1 deficiency affects the VIP+ neurons in the SCN core region

**FIGURE 7:**
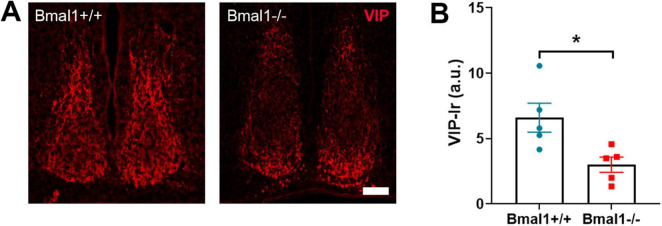
Bmal1 deficiency affects the vasoactive intestinal peptide (VIP) immunoreaction in the suprachiasmatic nucleus (SCN). **(A)** representative photomicrographs showing immunoreaction (Ir) of VIP (red) in SCN core region in Bmal1+/+ and Bmal1–/– mice. Scale bar = 100 μm. **(B)** Quantification of the VIP-Ir in the SCN. Unpaired-*t*-test. *P < 0.05.

Finally, we analyzed the VIP neurons, which are the primary retinorecipient neuronal population of the glutamatergic retinal input to SCN. The immunoreaction of VIP was significantly decreased in the core region of SCN in Bmal1−/− compared to Bmal1+/+ (*P* = 0.02) (Figure 7). This suggests a lower activity of the retinorecipient neurons in the SCN.

## 4 Discussion

Signal transmission from the retina to the brain, which is glutamatergic, plays an important role not only in image formation but also in non-image-forming photic responses such as pupillary light response and acute suppression of locomotor activity by light (Hattar et al., 2002; Panda et al., 2002; Ruby et al., 2002; Berson et al., 2002; Panda et al., 2003; Mrosovsky and Hattar, 2003) as well as in mood, emotion and cognition (Lazzerini Ospri et al., 2024; Fernandez et al., 2018) . There is evidence that the clock gene Bmal1 is required not only for the generation of circadian rhythms (Bunger et al., 2000) but also for behavioral and molecular responses to light (Pfeffer et al., 2015; Bunger et al., 2000; Yang et al., 2019; Pfeffer et al., 2009) as well as visual image processing (Baba et al., 2018). In this study, we investigate how Bmal1 deficiency affects neuronal and glial glutamate transporters as well as the ultrastructure of the retina-SCN synapse. A better understanding of the role of the molecular clockwork in light transmission to the brain could also help better understand the alteration in brain function in response to chronodisruption.

Consistent with earlier studies (Pfeffer et al., 2015; Bunger et al., 2000), Bmal1−/− mice showed an impaired response of locomotor activity to the light-dark cycle. Consistent with a study on retina-specific deletion of Bmal1 (Baba et al., 2018), Bmal1−/− mice showed a thinner retina. However, the number of melanopsin-positive cells, representing the ipRGCs which are essential for non-image-forming photic responses (Hattar et al., 2002; Panda et al., 2002; Ruby et al., 2002; Berson et al., 2002; Panda et al., 2003; Mrosovsky and Hattar, 2003; Fernandez et al., 2018; Lazzerini Ospri et al., 2024), was not affected in Bmal1−/− mice. Therefore, it is unlikely that the impaired non-image-forming photic responses are due to morphological changes in the retina. However, immunoreaction of vGLUTs, which are essential for glutamatergic signaling (Engelund et al., 2010), in the retina was altered by Bmal1-deficiency. vGLUT1, which is essential for transmitting visual signals from rods and cones to bipolar and RGCs but dispensable for non-image-forming responses (Johnson et al., 2007) was upregulated in the OPL of Bmal1−/− mice. This may be a compensatory upregulation in response to thinning of the photoreceptor layer. Alternatively, it might be a consequence of the loss of retinal circadian clock function reported earlier in a retina-specific deletion of Bmal1 (Storch et al., 2007) because if the molecular clockwork is defective, the light-dependent fluctuation of vGLUT1 in the brain is abolished and shows a constantly high level (Yelamanchili et al., 2006). In contrast, vGLUT2, which is essential for non-image-forming responses to light (Gompf et al., 2015; Purrier et al., 2014; Land et al., 2004), was decreased in the RGC layer of Bmal1-deficient mice. Therefore, altered glutamatergic signaling from the bipolar cells to the RGCs could contribute to the attenuated non-image-forming photic responses of Bmal1−/− mice.

The ipRGCs transmit light information to a wide variety of subcortical and cortical brain regions. Among the subcortical regions that receive strong innervation from ipRGCs, the SCN is the most studied. Therefore, we examined the influence of Bmal1 deletion on the glutamate transporters in the SCN. Both vGLUT1 and vGLUT2 were detectable in the SCN, with vGLUT2-Ir being stronger, consistent with vGLUT2 being the preferred subtype in the ipRGCs (Engelund et al., 2010). In Bmal1−/− mice, both vGLUT1- and vGLUT2-Ir was reduced, supporting the hypothesis that the molecular clockwork plays a role in the control of glutamate transporters and thus glutamatergic synaptic efficacy (Darna et al., 2009; Chi-Castañeda and Ortega, 2018; Kiss et al., 2007; Yelamanchili et al., 2006).

We analyzed integrity of the synapses in the retino-recipient core SCN region at the ultrastructural level. There was no difference in the symmetric, and therefore putative inhibitory synapses, within SCN core between both genotypes. However, in the asymmetric, and therefore putative excitatory/glutamatergic synapses, the size of the presynaptic terminal and the distance of the vesicles to the synaptic cleft was larger in Bmal1−/− mice. In contrast to a study on the cerebral cortex in older Bmal1−/− mice with neurodegeneration (Musiek et al., 2013), we did not see a reduction in the number of synaptic vesicles. This discrepancy could be due to the neurodegeneration associated with accelerated aging or to differences in brain regions. Vesicles with a shorter distance to the presynaptic membrane are particularly important for the immediate release of transmitters by the readily releasable pool and thus synaptic strength (Kaeser and Regehr, 2017). The increased distance of the vesicles from the presynaptic membrane in Bmal1−/− mice may, hence, contribute to reduced synaptic efficacy (Kaeser and Regehr, 2017). Thus, our data suggests that the efficacy of the synapse between the ipRGC and the SCN neurons at the structural level is affected by the Bmal1-deletion. Furthermore, there is evidence that Bmal1 rhythmically regulates the synaptic plasticity in the hippocampus by modulating the activity of synaptic calmodulin kinase (CamKII*a*) (Barone et al., 2023), which is involved in maintaining an optimal range of glutamate release probability (Hinds et al., 2003). Almost all SCN neurons are GABAergic (Reghunandanan and Reghunandanan, 2006; Varadarajan et al., 2018; Wen et al., 2020). The glutamate is released in SCN mainly via RHT terminals (in combination with PACAP) in response to light during the day to mediate light-induced neuronal firing and by astrocytes during the night to increase the presynaptic GABA release and consequently suppress the postsynaptic neuronal activity (Brancaccio et al., 2019; Brancaccio et al., 2017; Reghunandanan and Reghunandanan, 2006). It is debatable that intrinsic glutamatergic neurons in SCN exist. On one hand, it has been shown that glutamate immunoreactivity was occasionally found in non-retinal terminals (de Vries et al., 1993). In addition, some glutamate-immunoreactive nerve cell bodies were identified in the ventral and dorsal SCN of Wistar rats (Hannibal et al., 2000). Also, glutamate was rhythmically released in the organotypic slice cultures of the rat SCN (Shinohara et al., 1998); however, it is not clear if the glutamate is of neuronal or astrocytic origin. On the other hand, using *in situ* hybridization data from the Allen Brain Institute,^2^ there was no expression of vGLUT1 and vGLUT2 mRNA (markers of glutamatergic neurons) in SCN.

Importantly, the SCN core receives other input fibers such as geniculo-hypothalamic tract (GHT) (Hundahl et al., 2012) which release contain NPY and GABA (Hundahl et al., 2010; Francois-Bellan et al., 1990). For a precise identification of RHT nerve terminals that target the VIP cells, colocalization of ipRGCs tracer and VIP staining at the synapses in future studies is of crucial significance.

Excitotoxicity is prevented both by a homeostatic autoregulatory mechanism that limits excessive glutamate release by presynaptic vesicles (Winter and Ueda, 2008; Daniels et al., 2004) and by astrocytic glutamate reuptake via GLAST (Todd and Hardingham, 2020). In the SCN of Bmal1-deficient mice, GLAST-Ir was significantly reduced and glutamate levels were significantly increased, indicating an insufficient glutamate reuptake mechanism. Therefore, the reduced vGLUT-Ir in the Bmal1-/- mice could be a consequence of the homeostatic response to excess glutamate. Our data are consistent with a reduction of GLAST and insufficient glutamate reuptake in clock gene-deficient cortical astrocytes (Beaulé et al., 2009). Similarly, in mice with conditional deletion of Bmal1 in GLAST-expressing astrocytes, glutamate levels in the cerebrospinal fluid are significantly increased (Barca-Mayo et al., 2020). These data suggest that the molecular clockwork plays a role in modulating extracellular glutamate availability and thereby also in preventing excitotoxicity in the central nervous system, which is implicated in the pathogenesis of neurodegenerative diseases (Cuellar-Santoyo et al., 2023). Increased excitotoxicity could contribute to progressive neurodegeneration, which is evident in older Bmal1−/− mice and has so far been mainly attributed to increased oxidative stress (Musiek et al., 2013). Interestingly, in the hippocampus, spine size is inversely correlated with the efficacy of local glutamate uptake and thus appears to determine the probability of synaptic crosstalk (Herde et al., 2020). Thus, the enlargement of the presynaptic terminals in the SCN of the Bmal1−/− mice could be related to the lower efficacy of glutamate uptake. Our previous data showed that glial synaptic coverage is reduced in the hippocampal mossy fiber synapses of young Bmal1−/− (Ali et al., 2020). It is, therefore, very likely that Bmal1 deficiency affects the structure-function relationships of the glutamatergic tripartite synapses in large parts of the brain.

In the SCN, VIP-neurons are excited in response to light, leading to release of VIP (Jones et al., 2018; Todd et al., 2020), which is crucial for rhythm robustness (Hamnett et al., 2019). We observed a decrease in VIP-Ir in Bmal1−/− mice, consistent with an impairment in behavioral response to light and rhythm stability and in line with our previous study, which showed that Bmal1 deletion affects the light-induced neuronal activity in SCN during day-time (Öztürk et al., 2021). We assume that this reduction is, at least partially, due to impaired glutamate transmission at the SCN tripartite synapse and hence, altered light input to VIP-neurons, as a consequence of a defect molecular clockwork in the neurons and astrocytes. Consistently, astrocytic Bmal1 deletion affects VIP-Ir (Barca-Mayo et al., 2017). On the other hand, VIP modulates astrocytic GLAST activity (Goursaud et al., 2008), indicating a reciprocal relationship between astrocytes and VIP-neurons in the SCN.

**FIGURE 8:**
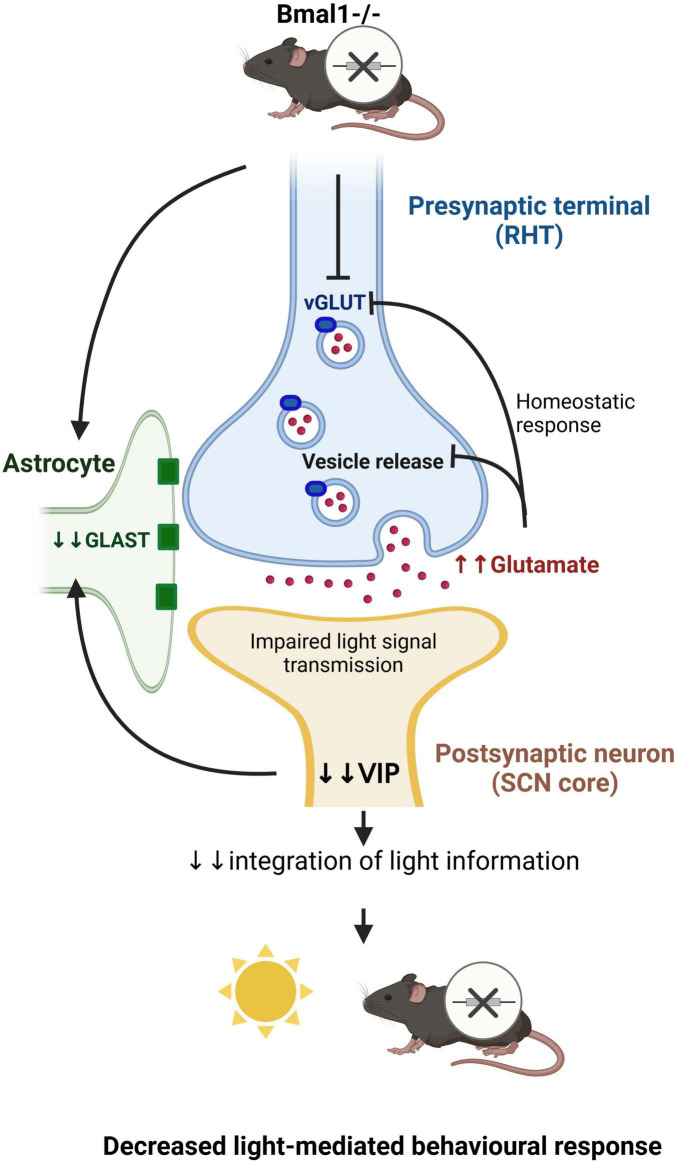
Schematic diagram showing that Bmal1-deletion affects the glutamate transmission at the tripartite synapse between the ipRGCs and the VIP-neurons in the SCN (Created in https://BioRender.com).

In conclusion, our data suggests that Bmal1-deletion affects the glutamate neurotransmission in the SCN (Figure 8). Further studies are needed to determine the molecular clockwork influence on other photic image-forming and non-image-forming brain functions in a similar manner. This would have far-reaching implications related to the modulation of various brain functions and possibly also increased excitotoxicity through chronodisruption.

## Data Availability

The datasets presented in this study can be found in online repositories. The names of the repository/repositories and accession number(s) can be found below: https://researchdata.hhu.de/handle/entry/180, 2024-12-03T13:41:42Z.

## Footnotes

1
http://rsb.info.nih.gov/ij

2
http://mouse.brain-map.org
