# Supplementary material for: Loss of Bmal1 impairs the glutamatergic light input to the SCN in mice
Source: Front Cell Neurosci. 2025 Feb 27;19:1538985. doi: 10.3389/fncel.2025.1538985 (PMC11903712; doi:10.3389/fncel.2025.1538985)
Supplement: Supplementary file 1 [file Data_Sheet_1.docx]

**Supplementary Figures**


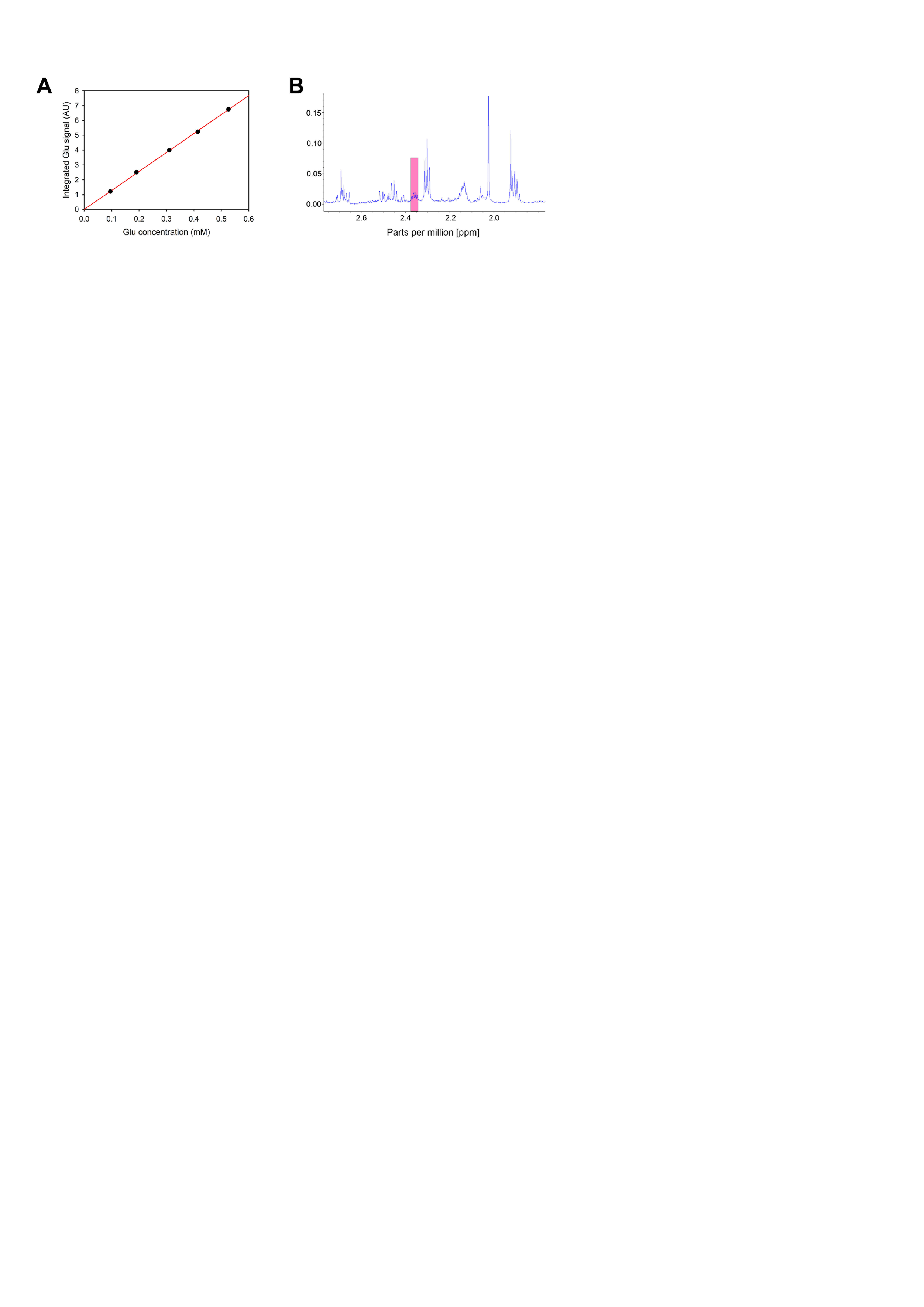


Supp. Figure 1. A) 1-dimensional ^1^H-NMR glutamate calibration curve. Standard calibration curve showing the linearity of integrated ^1^H-NMR signal intensity in the range of 95 to 526 µM glutamate in the NMR test tube. 1-dimensional ^1^H-NMR data in the range from 2.3776 - 2.3386 ppm (parts per million) were integrated and plotted against the concentrations of a glutamate standard. AU, arbitrary units. B) Glutamate determination in tissue extracts by 1-dimensional ^1^H-NMR. Glutamate concentrations of tissues were determined by 1-dimensional ^1^H-NMR. Here, as an example, the 1-dimensional ^1^H-NMR spectrum of a tissue sample obtained by dual phase metabolite extraction is shown. ^1^H-NMR signals characteristic for the CγH_2_ moiety of glutamate in the range from 2.3776 -2.3386 ppm (pink region) were integrated and the glutamate concentration were determined by using the ERETIC2 concentration calculation tool (TopSpin, Vers. 3.6.4). For the extract of a tissue sample, a glutamate concentration of 13 µM was determined.


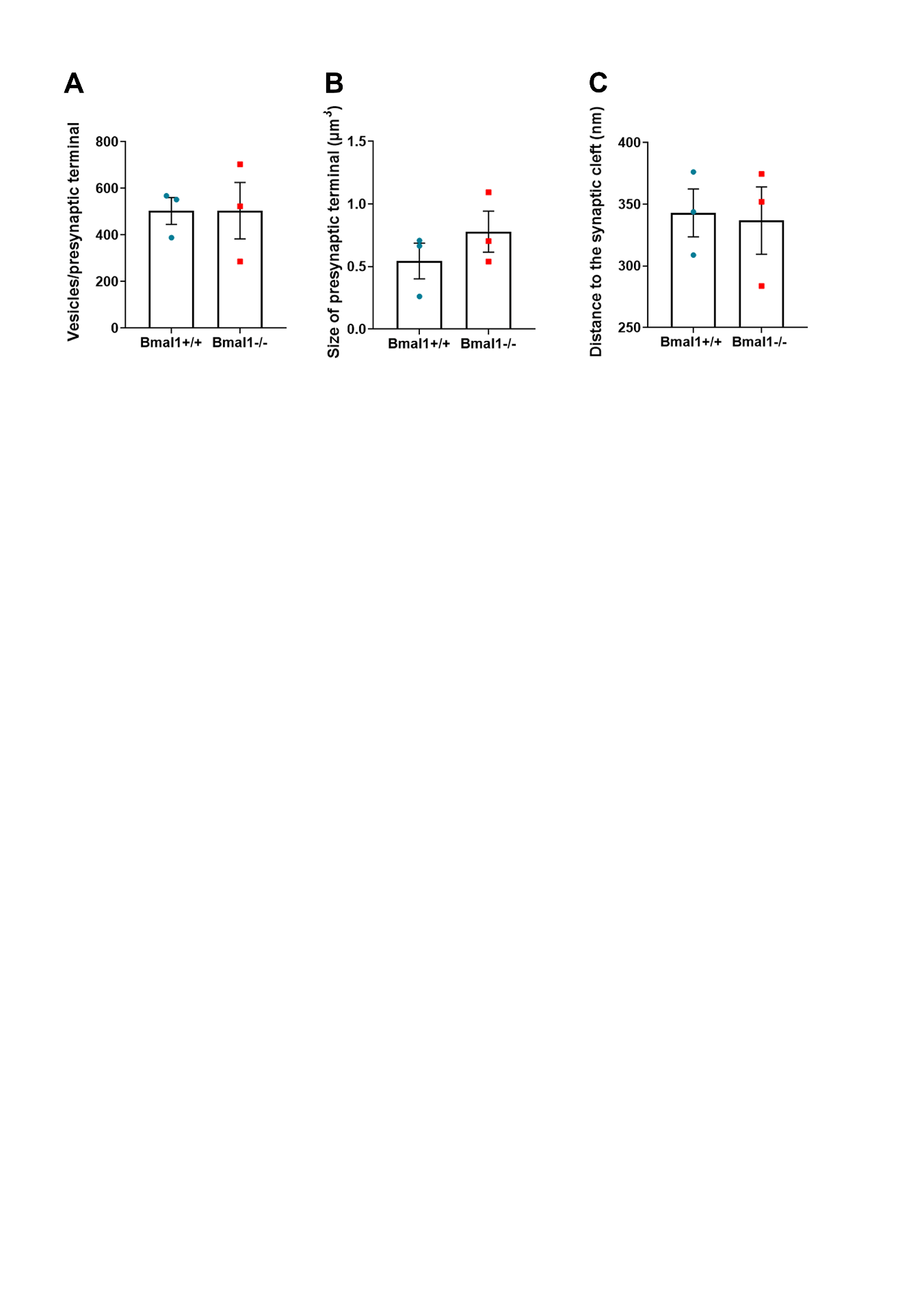


Supp. Figure 2. Bmal1 deficiency doesn’t affect the ultrastructure of presynaptic terminals of the symmetric/inhibitory synapses in SCN core regions. A) Quantification of the number of synaptic vesicles pro presynaptic terminal. B) Quantification of the size of the presynaptic terminals in µm^3^. C) Quantification of the mean distance between the synaptic vesicles and the synaptic cleft in nm. Unpaired-t-test, n= 3 mice per genotype.
